# Prediction of Total Anthocyanin Content in Single-Kernel Maize Using Spectral and Color Space Data Coupled with AutoML

**DOI:** 10.3390/s26030805

**Published:** 2026-01-25

**Authors:** Umut Songur, Sertuğ Fidan, Ezgi Alaca Yıldırım, Fatih Kahrıman, Ali Murat Tiryaki

**Affiliations:** 1 Department of Field Crops, Faculty of Agriculture, Çanakkale Onsekiz Mart University, 17100 Çanakkale, Türkiye; fkahriman@comu.edu.tr; 2 Department of Computer Science, Faculty of Engineering, Çanakkale Onsekiz Mart University, 17100 Çanakkale, Türkiye; fidansertug@gmail.com (S.F.); tiryaki@comu.edu.tr (A.M.T.); 3 Department of Biology, Faculty of Science, Dokuz Eylül University, 35160 İzmir, Türkiye; ezgi.alacaa@gmail.com

**Keywords:** plant pigments, near infrared reflectance, machine learning, *Zea mays*

## Abstract

The non-destructive and chemical-free determination of anthocyanin content in single maize kernels is of great importance for plant-breeding programs. Previous studies have mainly relied on Near-Infrared Reflectance (NIR) spectroscopy and color-based approaches, often using conventional or randomly selected modeling techniques. In this study, an Automated Machine Learning (AutoML) framework was employed to predict anthocyanin content using spectral and digital image data obtained from individual maize kernels measured in two orientations (embryo-up and embryo-down). Forty colored maize genotypes representing diverse phenotypic characteristics were analyzed. Digital images were acquired in RGB, HSV, and LAB color spaces, together with NIR spectral data, from a total of 200 kernels. Reference anthocyanin content was determined using a colorimetric method. Ten datasets were constructed by combining different color space and spectral features and were grouped according to kernel orientation. AutoML was used to evaluate nine machine learning algorithms, while Partial Least Squares Regression (PLSR) served as a classical benchmark method, resulting in the development of 1918 predictive models. Kernel orientation had a notable effect on model performance and outlier detection. The best predictions were obtained from the RGB dataset for embryo-up kernels and from the combined RGB+HSV+LAB+NIR dataset for embryo-down kernels. Overall, AutoML outperformed conventional modeling by automatically identifying optimal algorithms for specific data structures, demonstrating its potential as an efficient screening tool for anthocyanin content at the single-kernel level.

## 1. Introduction

Maize (*Zea mays* L.) is the world’s most widely produced hot-season cereal, playing an important role in human nourishment and as feedstuff. It has also gained an important place in various industrial fields with uses such as maize syrup, natural food coloring, and biofuel [1]. It is commonly grown in almost every region of the world because it is highly adaptable for different environments except cold areas. Maize, known to contain plenty of carbohydrates, fiber, vitamins, and minerals, is one of the plants providing the most carbohydrates in the world. In addition, high contents of secondary metabolites further expand the area of use and increase nutritional quality [2]. Colored maize genotypes especially have higher values in terms of secondary metabolites than common maize genotypes [3]. Colored maize is a special type of maize rich in anthocyanins and other phytochemicals. Anthocyanins are critical secondary compounds, and as water-soluble pigments, they cause a wide variety of colors in the plant [4]. Red, purple, and black are common colors of colored maize genotypes, and they are important alternatives to synthetic food coloring to produce natural dyes. Colored maize genotypes have also been proven to be beneficial for health [5]. With increasing health problems worldwide, people have begun to demand foods with high bioactive content. Colored maize has attracted increasing attention due to high phenolic compounds [6]. The antioxidant capacity of colored maize populations is higher than that of white maize populations [7]. In addition, it has positive effects on the fight against diseases such as obesity [8], diabetes [9], and cancer [10]. Due to these positive characteristics, different breeding programs are being carried out to develop colored maize lines. To achieve breeding program objectives, it is essential to employ non-invasive techniques that assess sample pigment content without kernel damage, aiding in material screening and selection. Analyzing anthocyanins without damaging the sample at the single-seed level is critical, especially for breeding programs to develop colored maize. Although destructive techniques such as wet chemistry and chromatography are used for this purpose, modeling approaches supported by spectral measurements and image analysis at the single-seed level are preferred. Numerous studies were conducted in the scientific literature to determine secondary metabolite content or classify samples based on image analysis [11]. However, studies about secondary compound determination using a combination of image and spectral analysis in maize are limited. Research carried out by Suriano et al. [3] investigates phenolic, total polyphenol, anthocyanin, and tocol compounds in maize using color space data. Four different colored genotypes were used in their study, and their research showed 91% success in separating maize varieties and different chemical components. Şerment and Kahrıman [2] reported that anthocyanin content could be detected with 96% accuracy based on spectral measurements only in milled samples between 1200 and 2400 nm. Mangalvedhe et al. [12] reported that LAB color space alone was insufficient to determine the total anthocyanin content (TAC); however, the model created based on Near-Infrared Reflectance (NIR) measurements could be used for rough screening purposes. Although there are studies in the literature about determining the anthocyanin content of maize samples by using color space data and spectral data separately, no study was found addressing whether anthocyanin content can be determined by using both color space and spectral data together at single-kernel level.

Studies on single-kernel analyses in the literature reported that different results were obtained from spectral measurements taken from the embryo-up and embryo-down positions of the maize kernel [13,14]. Moreover, the side of the kernel in which spectral data is collected affects the model’s success for some biochemical components, such as oil content [15]. The impact of seed orientation on the efficacy of predictive models for biochemical compound assessment, utilizing both digital imagery and spectral data from the embryo-down (opposite the embryo) and embryo-up (side with the embryo) positions, presents an intriguing research avenue. This interest arises from the potential variations in light absorption and reflection, pigment distribution, and spectral signature, which could significantly influence model accuracy and reliability. Understanding these discrepancies provides insights into image analysis techniques and spectral data interpretation for biochemical compound quantification. Given the variation in results for detecting biochemical components from data gathered in both embryo-up and embryo-down positions, a comparative study focusing on anthocyanin levels in these two positions is warranted. The variation in results can be attributed to the presence or absence of color pigments in the aleurone and pericarp layers, which can influence the perceived color intensity and pigmentation in the embryo region [16]. This variation has implications for both spectral analysis and digital imaging data. In the current literature, there are some studies emphasizing that the biochemical content of maize grain can be successfully determined by NIR [17,18]. However, in most of these studies, classical modeling techniques such as PLSR have been used. One of the major problems of single-seed analyses in species such as maize, where seed surface and biochemical content are not homogeneous, is that classical modeling techniques are inadequate to explain the non-linear relationship between light and matter. In order to overcome this problem, modeling techniques such as machine learning/deep learning, which can better explain the non-linear relationships between light and matter, have started to be used in scientific studies or applied fields. Machine learning has a wide range of applications in plant research, including seed classification [19,20], seed weight estimation [21], and the detection of plant diseases [22]. Additionally, studies have explored the determination of anthocyanin content in various plant species. For instance, machine learning was employed to estimate anthocyanin content in prunus plant leaves, achieving a prediction error of 0.34 mg/kg [23]. Similarly, anthocyanin levels in the leaves of winter wheat at different developmental stages were assessed using machine learning coupled with hyperspectral data, with the best model achieving an R^2^ value of 0.95 [24]. To date, no study has reported the detection of anthocyanin in single intact maize grains. However, anthocyanin content in maize leaves has been estimated using hyperspectral imaging combined with machine learning, where the most successful model achieved an R^2^ value of 0.868 [25]. One of the most important problems in this field is which of the many different approaches known as machine learning techniques should be used for model development [26]. The AutoML techniques include Auto-Keras, Auto-PyTorch, Auto-Sklearn, AutoGluon, H_2_O AutoML, rminer, TPOT, and TransmogrifAI [27]. Automated Machine Learning (AutoML), Feurer et al. [28], offers a batch approach that lets us decide which machine learning algorithm to use on a given dataset, whether and how to preprocess its features, and how to set all hyperparameters. AutoML has wide variability for machine learning techniques such as Decision Trees, LightGBM, XGBoost, CatBoost, Neural Networks, Random Forest, Extra Trees, sSacked, Golden Features, Selected Features, Ensemble, Ensemble Stacked. The method discussed enables the evaluation of various machine learning techniques to determine which has high modeling success, while also selecting variables that enhance predictive power. It includes an option for “golden feature selection,” where a “golden feature” significantly improves model performance. In contrast, “selected features” are determined through a feature selection process, focusing on improving predictive accuracy and relevance. An effective machine learning workflow combines domain expertise to identify golden features and systematic feature selection for simplicity and interpretability. This approach is well-suited for developing NIR prediction models and color space datasets based on machine learning. Notably, there are no existing studies applying this technique to NIR and image data from different sample positions for determining anthocyanin content in single maize seeds using AutoML.

The aim of this study was to develop and compare prediction models based on the classical (PLSR) and AutoML approach for the determination of total anthocyanin content using different combinations of color space data and spectral data extracted from digital images of single-kernel samples, with the additional objective of identifying the most effective modeling strategy under limited sample size conditions. Differences in color spaces and spectral measurements of colored maize samples were also evaluated, and the effect of seed position (embryo-up/embryo-down) on the results of modeling studies is discussed.

## 2. Materials and Methods

### 2.1. Plant Material

A total of 40 different genotypes, including 38 colored genotypes and 2 standard hybrid varieties, were used as material in the current study (Table A1). A total of 200 seed samples, with a random selection of five seeds from each genotype, were studied. Some of the genotypes used in the study were previously screened for anthocyanin content within the scope of scientific research at Çanakkale Onsekiz Mart University, Faculty of Agriculture, Department of Field Crops. The rest of the genotypes were obtained from the Eastern Mediterranean Agricultural Research Institute. The genotypes have the characteristics of dent, flint, popcorn, and sweet endosperm, which have purple, black, red, orange, yellow, and white kernel colors. Kernels were cleaned first then sorted and labeled with unique codes. These codes were saved in an Excel file and used in further analyses.

### 2.2. Image Data Extraction and Acquisition of NIR Data

Samples were scanned using a commercial desktop scanner (HP 3970, HP Development Company, LP, Spring, TX, USA) at 300 dpi resolution. Scanning was performed for “embryo up”, where the embryos of the seeds were facing the scanner surface, and “embryo down”, where the embryos of the seeds were positioned opposite to the scanner surface. This was performed in such a way that the order of the seeds was not mixed. Image processing was performed with the R program (version 4.3.1) [29]. In this context, edge detection and color space channel data were extracted from jpeg files using EBImage (version version 4.14.2) and colorspace (version 2.1-2) packages [30,31]. For color space data, mean, standard deviation, skewness, kurtosis, and median values of RGB, HSV, and LAB color spaces were determined. These data, extracted at the single-kernel level for each color space, were saved with previously labeled unique codes to be used as predictor variables.

NIR spectroscopy scans were performed at the single-kernel level using the SpectraStar 2400D (Unity Scientific, Milford, MA, USA), covering a wavelength range of 1200–2400 nm with 1 nm resolution. Each kernel was scanned in both the embryo-up and embryo-down orientations, taking the average spectrum from 48 scans per orientation. When obtaining these data, measurements were made using a sample container suitable for single-seed measurement specific to the NIR spectroscopy device. To prevent confusing the order of the imaged kernels, the unique codes that were previously determined in the labeling step were used.

### 2.3. Total Anthocyanin Content (TAC) Determination

Anthocyanin determination was carried out by adapting the study of Abdel-Aal and Hucl (1999) [32]. Before analysis, single-seed samples were ground using liquid nitrogen. For total anthocyanin determination, 0.8 mL of 0.1 N methanolic HCl (St. Louis, MO, USA) was added to 100 mg samples, shaken for 30 min at room temperature, and then centrifuged (Hettich|North America,  Beverly, MA, USA) at 4500× *g* for 20 min. After that, 100 µL of sample extract and 100 µL of methanolic HCl taken from the supernatant were placed in a 96-well plate with 3 replicates. The wavelength of the samples was measured at 535 nm using a microplate reader (Agilent, BioTek, Santa Clara, CA, USA). TAC was determined according to the equation proposed by Abdel Aal and Hucl [32].

### 2.4. Datasets Preparation and Data Preprocessing

In this study, a total of 10 datasets were utilized, divided into two main groups according to the embryo positions of the seeds. These main groups are “embryo-up” and “embryo-down”. Each group consists of five datasets, representing different color spaces and sensor data: RGB, HSV, LAB, Near-Infrared (NIR), and a combination dataset integrating all these four data types.

To ensure the integrity and quality of the datasets, Principal Component Analysis (PCA) was initially applied to the combination datasets of both “embryo-up” and “embryo-down” groups. This process was performed to improve datasets by identifying and eliminating outliers. Outliers were identified and eliminated separately from the embryo-up and embryo-down datasets based on the RobustPCA algorithm. The rospca package (version 1.1.1) developed in the R program (version 4.3.1) was used for this purpose [33]. The PCA process resulted in datasets comprising 181 samples for the “embryo-up” position and 185 samples for the “embryo-down” position from a total of 200 samples.

For the generation of new specific datasets, the following procedure was applied for each case, including RGB, HSV, LAB, NIR, and the combination datasets: For each color space-based dataset, the corresponding values (e.g., red mean, red_stdev, red median, green mean, green_stdev, green median, blue mean, blue_stdev, blue median values for the RGB dataset) were extracted from the combination datasets. The output variable (anthocyanin content) was integrated into the dataset alongside the extracted features. A new dataframe was constructed containing both the feature values and the output variable. The dataset was normalized using Min-Max Scaling, a process that scales the features to a fixed range, typically 0 to 1 by excluding the output variable. After normalization, the output variable was reincorporated into the dataframe, completing the dataset preparation process for each specific data type. This data generation procedure, applied uniformly to all datasets, ensured consistency across all datasets and facilitated analysis of the effect of different data types and embryo positions on the observed outcomes. Through preprocessing and normalization processes, the datasets maintained high data quality and ensured reliable results in subsequent analyses.

### 2.5. Development and Evaluation of Prediction Models

In the study, two different modeling methods were utilized: one used PLSR as a classical technique, while the other employed an AutoML approach. Throughout the analysis, a total of 1918 predictive models were developed across all datasets (Table 1). The number of variables in the dataset was changed by data used. Table 1 serves as a concise yet comprehensive overview of the datasets involved in the model training process. Each row corresponds to a specific version or type of dataset, capturing its essential characteristics—the number of rows and columns and how many models are created for that specific dataset.

We have utilized Partial Least Squares Regression (PLSR) models with input data A 5-fold cross-validation approach was employed to ensure the robustness and reliability of the models. To determine the optimal number of components for each dataset, comprehensive analyses were conducted. The maximum number of components was set as follows: nine for the HSV, RGB, and LAB-based datasets, 40 for the NIR-based dataset, and 60 for the dataset consisting of the combined features of all datasets. Through rigorous experimentation, the component numbers that minimized the Mean Squared Error (MSE) for each dataset were identified and subsequently used for model training. For the embryo-down position, the optimal component numbers were found to be 3 for the combined dataset, 3 for HSV, 4 for LAB, 9 for NIR, and 5 for RGB. Similarly, for the embryo-up position, the optimal components were determined as 4 for the combined dataset, 2 for HSV, 3 for LAB, 7 for NIR, and 4 for RGB.

The expected and predicted regression lines for the different datasets were plotted. These analyses and visualizations guided the development of robust PLSR-based models, tailored specifically for each type of dataset considered in this study.

During the model training and evaluation process firstly, each dataset was input separately into the MLJAR-supervised AutoML framework (referred to as “mljar_supervised” by Płońska and Płoński, 2021) [34]. The MLJAR framework’s “Compete” mode was employed, which automates the machine learning process by testing a range of models and configurations to identify the most effective approach for the given task.

The training process incorporated advanced feature engineering and selection techniques, such as the construction of golden features and feature selection methods, to enhance the predictive performance of the models. Nine distinct algorithms were evaluated as part of the model selection process. These algorithms are Decision Trees, Linear Model, Random Forest, Extra Trees, LightGBM, XGBoost, CatBoost, Neural Networks, and Nearest Neighbors.

We have used the default hyperparameter search space provided by the mljar-supervised framework [34]. When training models in compete mode, the library explores the following hyperparameter configurations:

Decision Trees were evaluated with criterion set to squared error and friedman_mse, while max_depth varied across 2, 3, and 4. XGBoost models utilized reg:squarederror as the objective function with max_depth ranging from 4 to 9. Default parameters included eta at 0.075, min_child_weight at 1, subsample at 1.0, colsample_bytree at 1.0, max_rounds at 10,000, and early_stopping_rounds at 50. CatBoost Regressor explored three loss functions (RMSE, MAE, and MAPE) with depth set to 6, learning_rate at 0.1, and rsm at 1.

For ensemble methods, both Extra Trees and Random Forest were configured with squared error as the criterion, max_features ranging from 0.5 to 1.0 (in 0.1 increments), min_samples_split varying across 10, 20, 30, 40, and 50, and max_depth spanning 3 to 7. Finally, Neural Networks were tested with dense_1_size set to 16, 32, or 64 neurons, dense_2_size set to 4, 8, 16, or 32 neurons, and learning_rate varying among 0.01, 0.05, 0.08, and 0.1.

All parameters not explicitly mentioned were maintained at their default values as specified in the respective scikit-learn, XGBoost, and CatBoost libraries.

For extra trees while training on compete mode, library sets criterion as squared error, max_features as 0.5, 0.6, 0.7, 0.8, 0.9, 1.0, min_samples_split as 10, 20, 30, 40, 50, and max_depth as 3, 4, 5, 6, 7. All other parameters are set as default scikit-learn variables.

For nearest neighbors while training on compete mode, the library sets n_neighbors as 3, 5, 7 and weights as uniform, distance. All other parameters are set as default scikit-learn variables.

To increase the robustness and accuracy of the predictive models, AutoML was configured to implement model stacking and ensemble learning techniques, which combine predictions from multiple models to reduce variance and improve generalization.

To ensure the reliability of the model evaluation, a stratified 5-fold cross-validation was employed. This approach involves randomly shuffling samples before dividing them into five subsets, allowing each subset to serve as both training and validation set across different iterations. This method helps mitigate overfitting and provides a more unbiased estimation of model performance.

The evaluation metric was selected automatically by the MLJAR framework, which identified Root Mean Squared Error (RMSE) as the most suitable measure of model accuracy. RMSE is a standard metric for evaluating the predictive accuracy of regression models, providing insight into the average deviation in predictions from true values. The evaluation section includes the formula used to calculate the RMSE.

A computational time limit of 18,000 s was allocated to the model generation process for each dataset. This constraint ensured that the analysis would be both exhaustive in exploring different model configurations and efficient in terms of resource utilization.

Through this comprehensive modeling strategy, the study aimed to derive insights into the effectiveness of various algorithms and techniques in predicting outcomes based on the provided datasets, and to identify the optimal model configurations tailored to the specific characteristics of each dataset.

The evaluation metrics used in this study are as follows [35]:

Mean Absolute Error (MAE): It is the mean of the absolute differences between the observed values and the predicted values by the model. It quantifies the average magnitude of errors in a set of predictions, without considering their direction.

Root Mean Squared Error (RMSE): This metric is the square root of the average of the squared differences between observed values and the predicted values. RMSE gives higher weight to larger errors, making it sensitive to outliers.

Mean Squared Error (MSE): Similar to RMSE but without the square root, it measures the average squared difference between the observed and predicted values.

R-squared (R^2^): This is a measure of the proportion of variance in the dependent variable that is predictable from the independent variable.

Mean Absolute Percentage Error (MAPE): This metric represents the Mean Absolute Error as a percentage of the observed values.

## 3. Results and Discussion

### 3.1. Changes in Color Spaces and Spectral Measurements According to Embryo-Up and Embryo-Down Sides

**Figure 1:**
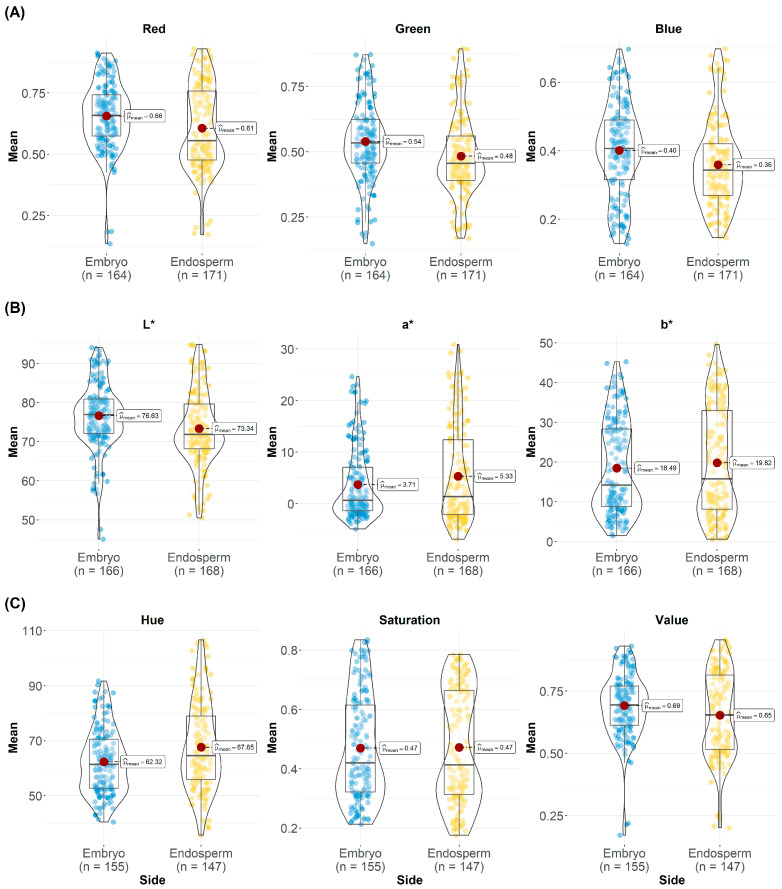
Differences in mean pixel values for the color space channels ((**A**): RGB Chanel, (**B**): Lab, (**C**): HSV).

Color space data extracted from images taken from embryo-up and embryo-down sides in different color spaces are shown in Figure 1. Some characteristic differences occur in the digital data from embryo-up and embryo-down depending on the channels of the color spaces. For example, the averages of R, G, and B channels obtained from embryo-down images in all RGB color space channels were lower than from the embryo-up side. This situation may be associated with the level of coloration on the embryo-up and embryo-down sides of the seeds. In our study, except for Maize Morado and samples with red kernel color, almost all other genotypes do not have full coloration of the pericarp layer of the embryo part of kernels. This situation depends on whether the compounds that cause pigmentation are present in both the aleurone and pericarp layers or only in the aleurone layer [36]. Thus, the light absorption of pigmented samples in a single layer may be higher than others, which results in an increase in the channel averages for the RGB embryo-up images. Hence, these evaluations coincide with the results obtained from the channel data of the LAB color space. The L* channel in the LAB color space represents darkness, where 0 is perfect black, a 50% rating with 0% reflectance or transmittance indicates medium gray, and a rating of 100 indicates perfect white. In our study, the average L channel value of embryo-up images was higher than embryo-down images. This can be attributed to the relatively lower number of samples containing pigmentation in the double layer, as described above. In the a* and b* channels, the averages of the channel data for embryo-down images were found to be higher than the embryo-up images. The a* channel in the LAB color space represents red-greenness of the color, while positive values of a* represent redness and negative values represent greenness. The level of 0 indicates neutral. The b* channel expresses the yellow-blueness of the color, and positive values of b* are yellow, negative values are blue, and 0 indicates neutrality. The high a* channel in the embryo-down images can be attributed to the high number of red-colored samples in the genotypes used in the study, since 10 of 40 samples had red-colored kernel characteristics.

HSV color space defines color with the terms hue, saturation, and value. Although a mixture of colors is used in RGB, hue, saturation, and brightness values are used in HSV. Saturation determines the vividness of the color, while brightness expresses the brightness of the color. For example, in HSV space, color, and saturation values for black can have any value between 0 and 255, while the brightness value will be zero. For white color, the brightness value is 255. In our study, the average color value (hue) data for this color space on embryo-down side images were higher than embryo-up side images. While the saturation (S) value had a close average for both sides, the brightness value (value) had a lower average on the embryo-down side.

It is understood that more comprehensive information about the color characteristics of the samples can be obtained if color spaces are used alone or together for single-kernel imaging studies. By looking at the channel data in the color space, it seems possible to understand the distribution of the color properties of the samples and the density and composition of the compounds that cause pigmentation in the outer part of the maize samples.

**Figure 2:**
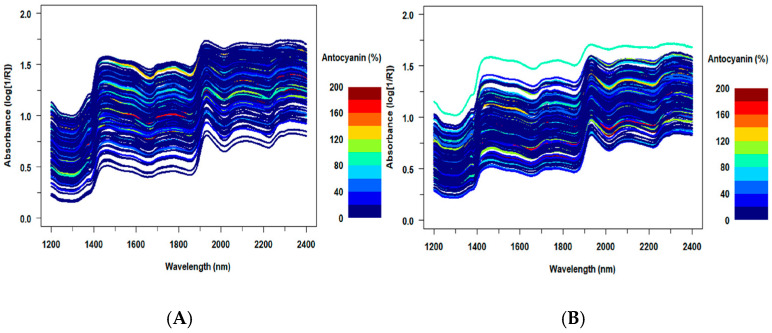
Spectral plot showing changes in anthocyanin content from embryo-down (**A**) and embryo-up (**B**) sides of single-seed samples.

**Figure 3:**
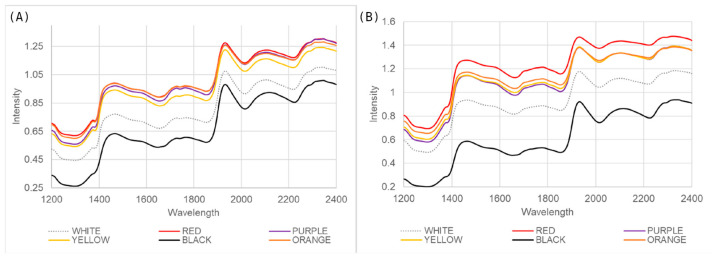
Average spectra for samples with different kernel colors from embryo-up (**A**) and embryo-down (**B**) sides of single-seed samples.

The changes in anthocyanin content for all samples in spectral data collected from the embryo-up and embryo-down sides are shown in Figure 2. There was no explicit grouping in the spectral data in response to the change in anthocyanin content over the whole dataset. On the other hand, significant differences were observed in the graph of the average spectra for six different color groups (black, purple, red, yellow, orange, white) used in the study (Figure 3). In the spectral data taken from both sides, the averages of black- and white-colored samples are clearly separated from seed samples with other colors. Black seeds have significantly lower spectral intensity values than the other groups throughout the measured spectral range. The mean values for yellow, orange, red, and purple genotypes are higher and generally show a similar spectral trend. Depending on the seed color, differences in spectral intensity values can be seen. In terms of spectral reflectance values of yellow, brown and black rapeseed seeds, dark-colored seeds had lower spectral reflectance values than light-colored seeds [37]. Similarly, the spectral reflectance value of the seeds of black maize genotypes was found to be lower than the other colored seeds in our study, as seen in Figure 3.

### 3.2. Evaluation of Prediction Models for Image and Spectral Datasets According to Embryo and Endosperm Side

Changes in the reference values of the samples used in modeling studies and the limits of these values greatly impact model success and evaluation parameters. Descriptive statistics of the samples used in the calibration models developed in this study, with the data collected in embryo-up and embryo-down directions, are presented in Table 2. The anthocyanin contents of the samples used in models created with the data obtained from the embryo-up position ranged from 5.53 mg/kg to 184.67 mg/kg. For the embryo-down position, values were between 5.53 mg/kg and 184.63 mg/kg in the samples used. The quantitative analysis of anthocyanin levels in samples obtained from the embryo-up orientation exhibited comparable results across the different models employed in this study. This directly affects the descriptive statistics for the anthocyanin content of the samples used in these models. The anthocyanin content in the maize kernel is influenced by the pericarp characteristics and the kernel color of the respective genotypes. Research investigating anthocyanin variations across maize genotypes with distinct kernel colors documented anthocyanin levels ranging from 15.4 mg/kg to 696.07 mg/kg [3,38,39,40,41]. Conversely, Salinas Moreno et al. [42] discovered that the anthocyanin concentration in the pericarp of purple and red maize genotypes was nearly 10-times higher than that in the entire grain, with a marked decrease in anthocyanin levels observed in the endosperm once the pericarp was removed. This indicates that the pericarp region harbors the highest concentration of anthocyanin. The thickness of the pericarp is a significant quality indicator for maize genotypes featuring colored grains. Although our research did not assess pericarp thickness, the genotypes examined may vary in this respect, potentially influencing alterations in both NIR and image data, as well as anthocyanin content. To elucidate these observations, further studies incorporating histological assessments are warranted.

For both embryo-up and embryo-down positions, ensemble-based models obtained the lowest values in terms of RMSE. While creating ensembles, multiple machine learning models have been integrated, each assigned a specific weight that reflects its contribution to the final prediction. The weighted approach ensures that models with higher predictive accuracies exert more influence on the ensemble’s output, while those with lower accuracies contribute less. For embryo-up RGB-based data and for embryo-down side combined data (RGB+LAB+HSV+NIR) created the best ensembles in terms of MSE.

**Figure 4:**
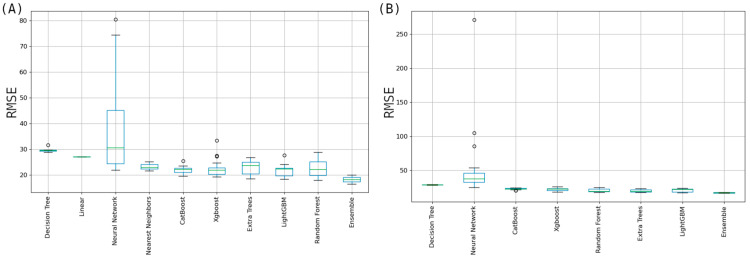
RMSE values for the models generated by AutoML approach for embryo-up (**A**) and combined data set on embryo-down (**B**) positions.

**Figure 5:**
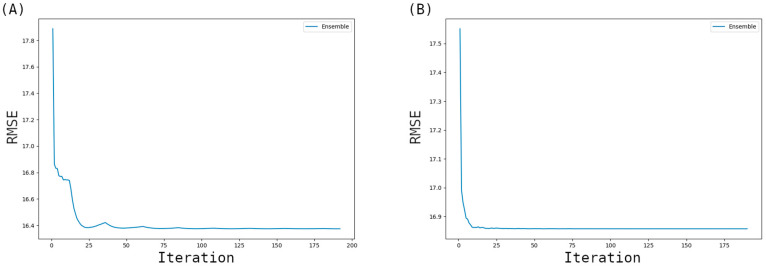
Changes in RMSE values by iterations for the best models for embryo-up (**A**) position which uses RGB dataset and embryo-down (**B**) position which uses HSV dataset.

For embryo-up position, RMSE statistics of the nine modeling methods based on the RGB dataset, developed using the AutoML approach, are presented in Figure 4A. As seen in the figure, it was determined that with the exception of the Neural Network model, the errors of the other models generally remained below 30 mg/kg. We believe that the reason for this situation is that the AutoML approach, in some runs, produced a very complex neural network model structure compared to the data we had during the experiments and that caused overfitting in some models due to complexity. The iteration graph of the ensemble model for embryo-up position (Figure 5A) which uses RGB dataset indicates that the RMSE value stabilizes and shows no significant change after approximately the 40th iteration. An examination of the sublayers included in the ensemble model revealed contributions from Random Forest Stacked (Weight = 82), Extra Tree Selected Stacked (Weight = 70), and Random Forest Selected Feature Stacked (Weight = 20), among others, with varying weight values contributing to the model’s performance.

The models in this ensemble include ExtraTrees, LightGBM, Neural Networks, and Random Forests, each contributing uniquely to the ensemble’s overall decision-making process. The RandomForest_Stacked model is the most significant contributor to this ensemble, with the highest weight of 82. Random Forests are known for their robustness in handling large datasets and their ability to capture complex interactions between features [43]. This large weight suggests that the RandomForest_Stacked model consistently provides reliable and accurate predictions, which significantly influence the ensemble’s final output. The ExtraTrees_SelectedFeatures_Stacked model holds the second-highest weight at 70. ExtraTrees is a variant of the Random Forest algorithm that introduces more randomness during tree construction. By utilizing selected features, this model can enhance its focus on the most informative variables, thus improving predictive performance. This substantial weight suggests ExtraTrees’ ability to complement the RandomForest model by capturing different patterns or aspects of the data. The remaining models, collectively, have lower weights, but they play crucial roles in diversifying the ensemble’s predictive power. Even though these models have relatively lower weights, their presence ensures that the ensemble can cater to a wider range of data patterns, ultimately leading to improved generalization and robustness in the ensemble’s predictions.

Figure 4B displays the RMSE results for high-performing AutoML models associated with embryo-down datasets which uses combined (HSV, RGB, NIR, LAB) datasets. In contrast, linear regression algorithms are not observed in the embryo-up scenario (Figure 4A). This discrepancy arises due to the AutoML system’s functionality, which disables linear regression when the dataset column numbers exceed the number of 1000 thereby impeding reliable ensemble model development. 

The MLJAR-Supervised library implements automatic constraints for certain algorithms to optimize computational efficiency. Due to the merged dataset containing 1228 columns, the library disabled the KNN (K-Nearest Neighbors) algorithm. MLJAR-Supervised library has set a maximum column limit of 100 (‘max_cols_limit: 100’) for the KNN algorithm; when this threshold is exceeded, the corresponding algorithm is automatically excluded from the training process.

Similarly, the Linear algorithm is also subject to constraints based on dataset size. The library disables the Linear algorithm when the number of rows exceeds 10,000 or the number of features exceeds 1,000. Therefore, Figure 4B contains two fewer algorithms compared to Figure 4A. As shown in Figure 4B, most models, except the Neural Network, successfully maintained error values below 30 mg/kg. The iteration graph of the embryo-down ensemble model (Figure 5B) which uses HSV dataset demonstrates that the MSE value (<17 mg/kg) showed no significant change beyond the 35th iteration. This finding suggests that the model stabilized early in the training process and completed its learning phase effectively. An analysis of the components within the ensemble model revealed that models such as LightGB_Stacked (Weight = 54), Extra_Trees_GoldenFeatures_SelectedFeatures_Stacked_Stacked (Weight = 40), and Random Forest GoldenFeatures Selected Features Stacked (Weight = 33) held significant weight within the ensemble model.

Among the models produced for the embryo-down side, again the ensemble has achieved the most efficient results in terms of RMSE. The dominant model in this ensemble is LightGBM_Stacked, which carries the highest weight of 54. LightGBM is a high-performance framework known for its efficiency and speed, particularly when working with large datasets [44]. Its ability to handle complex interactions between features while maintaining computational efficiency makes it an essential component of this ensemble, allowing it to heavily influence predictions with its precise results. LightGBM’s advanced boosting algorithm ensures that it captures a wide array of patterns in the data, which explains the significant weight assigned to it, marking it as a cornerstone. Another crucial element in this ensemble is the Extra_Trees_GoldenFeatures_SelectedFeatures_Stacked model, manifested twice with cumulative weights of 81 (40 and 41). This model, a variant of the Extra Trees algorithm, enhances the ensemble’s capacity to explore and leverage feature interactions effectively. By incorporating “golden” and “selected” features, this model taps into a curated subset of particularly informative features, allowing it to improve precision and avoid noise. The substantial cumulative weight assigned to these models in the ensemble suggests their success in capturing unique patterns that complement the LightGBM component. The RandomForest_GoldenFeatures_Selected Features_Stacked model, with a weight of 33, emphasizes the importance of feature engineering and selection—a theme present throughout the ensemble. Random Forest models are particularly valued for their adaptability and relatively robust performance across different datasets without extensive parameter tuning [45]. By using “golden” and “selected” features, this model likely addresses key areas of prediction that are missed by others. The rest of models, despite the lower weights, adds an additional layer of flexibility and breadth to the ensemble’s predictive capabilities, making it adept at managing a wide variety of data structures and patterns.

The evaluation parameters of the best from AutoML and classical (PLSR) models for determining anthocyanin content using digital images and spectral data collected from the embryo-up and embryo-down sides are presented in Table 3.

For the embryo-up position, the models based on PLSR, which is the classical modeling technique, had the highest modeling error (MAE of 23.81) using the NIR dataset and exhibited a mean MAE of 20.61. The average R^2^ value was 0.28, which indicates limited predictive power with the classical approach. AutoML approach significantly improved predictions for embryo-up position by reducing average MAE to 12.47. Best results were noted by the RGB dataset (MAE of 10.33) and RGB+LAB+HSV+NIR (MAE of 10.78) dataset. This approach showed a strong overall R^2^ value of 0.66, showing enhanced predictive accuracy over the classical method (Table 3).

For the embryo-down position, classical (PLSR)-based models had the higher modeling errors (MAE of 21.29) using NIR dataset and (MAE of 17.77) RGB datasets. Classical approach exhibited a mean MAE of 17.71. In PLSR models, average R^2^ value had a modest improvement (0.39) compared to the embryo-up position. Again, the AutoML approach had notable improvement with a mean MAE of 11.16. Best results were noted by the HSV dataset (MAE of 9.66, R^2^ of 0.75) and combined (MAE of 10.24, R^2^ of 0.76) dataset. This approach achieved an enhanced overall R^2^ value of 0.70, confirming its superior performance. In the context of AutoML methodology, similar outcomes were observed across certain evaluation metrics when comparing the HSV dataset with the combined dataset. Although the HSV dataset demonstrated greater efficiency in regard to MAE and MAPE metrics, the combined dataset showed superior performance in RMSE, R^2^, and MSE metrics. The results of an earlier study show that the R-squared metric is more informative, truthful and devoid of interpretability limitations of other metrics for regression analysis [35]. Hence, in our study, R-squared has been chosen as the preferred metric to evaluate the best model.

As a result, the combined dataset was determined to be the most effective choice.

**Figure 6:**
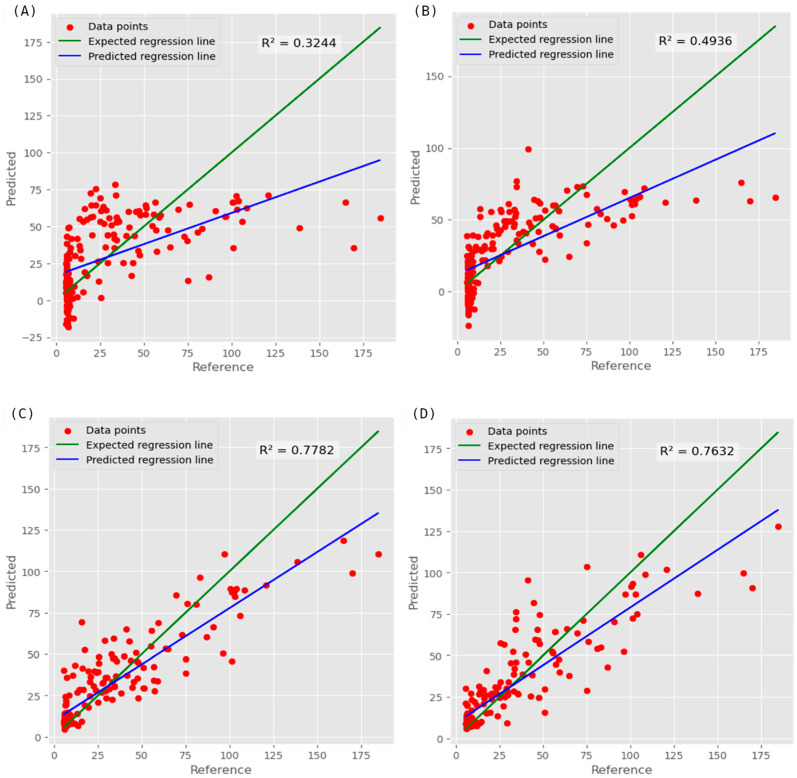
Scatter plot of observed–predicted values from the best prediction models generated by PLSR for embryo-up (**A**) and -down position, (**B**) and AutoML approach for embryo-up (**C**) and -down (**D**) position.

Ensemble models provided superior predictive accuracy compared to the PLSR models in both kernel positions, with particularly marked improvements in the embryo-down position when using the combined (RGB+HSV+LAB+NIR) dataset. The models developed for both seed orientations can be utilized for rough screening purposes in determining the anthocyanin content of single-kernel samples. For both embryo-up and embryo-down datasets, ensemble models that leverage their collective strengths by aggregating predictions from multiple algorithms to enhance overall performance and reliability consistently yielded the most positive results in terms of evaluation statistics and demonstrated superior prediction accuracy compared to their individual modeling approaches. The results highlight the critical role of ensemble modeling in achieving optimal predictive outcomes. Our results show that the AutoML approach consistently outperformed the classic PLSR model in all metrics across both embryo-up and -down positions (Figure 6). This demonstrates the robustness and effectiveness of the ensemble method in handling diverse datasets and improving model accuracy.

While no existing studies specifically investigate the quantification of anthocyanin content at the single-kernel level in maize by color data plus spectral measurements, research into the determination of anthocyanin levels in various plant species utilizing spectral and image data does exist [46]. Nankar et al. [47] reported R^2^ values ranging from 0.15 to 0.35 in their research on NIR detection of 143 distinct phytochemicals, including anthocyanins, in maize. Mangalvedhe et al. [12] observed R^2^ values between 0.72 and 0.93 in the calibration set for their PLSR models, which were developed using spectra from intact kernels in bulk. In a study using ground samples and applying spectral pre-treatment specifically for anthocyanin content, R^2^ values of up to 0.96 and 0.90 were achieved [2]. These findings underscore the significance of variables such as the state of the sample (intact seed versus ground) and the application of data pre-treatment for the successful detection of anthocyanins, particularly when using spectral data. Given that our study employed single-kernel samples, the spectral data may exhibit more variability compared to ground samples. Nonetheless, the outcomes of our investigation are in line with those from previous studies involving maize samples, suggesting comparability despite the noted differences, although there are some studies about predicting anthocyanin. In heterogeneous and intact biological samples such as seeds, the predictive performance of NIR-based models may be relatively limited due to within-sample variability, scattering-related spectral effects, measurement orientation, and the limited repeatability of reference methods. Nevertheless, the correlation and error metrics obtained under these challenging conditions are comparable to those reported in the literature for NIR-based prediction of phenolic and anthocyanin contents in other seeds and fruits. This indicates that the performance of the developed models is methodologically acceptable for systems characterized by high biological heterogeneity [48]. For content in maize by NIR spectroscopy, no study was identified that directly determines anthocyanin content in individual maize kernels based on image analyses and color spaces. However, it was reported that anthocyanin content in common bean seeds can be detected with an accuracy of 85–87% using HSI color space data and deep learning methods [49]. Our study did not achieve this level of success, which could be attributed to differences in the set of materials used and the model construction techniques employed. In this study, spectral data collection was also performed using a bench-top NIR device which is not suitable for contact measurement and cannot set sample–detector distance. This may affect the quality of the spectral data and its relevance to the nature of analyzing for anthocyanin content in intact maize grains.

## 4. Conclusions

The color attributes of maize kernels were assessed using digital image analysis. These variations could shed light on the specific pigment compositions responsible for color development and the resulting coloration types in maize. Models developed by utilizing color space data, both solely and in combination with data from single-seed NIR spectroscopy, exhibited varying levels of accuracy in predicting anthocyanin levels on a per-kernel basis. The position of kernels for collecting digital images and the NIR spectra was found to have influenced the efficiency of well-performing models. Among the models created using embryo-up measurements, the RGB color space yielded more successful results than the other models. Conversely, for embryo-down measurements, the combination of RGB+HSV+LAB+NIR proved to be the most successful. The models created for quantitative estimation of anthocyanin content for both seed directions were suitable for rough screening.

This study provides a representative framework for selecting appropriate modeling strategies prior to the application of machine learning and deep learning models based on single-kernel spectral and image data. The proposed approach may support more effective selection and discrimination in seed-breeding research as well as in industrial applications within the food sector, enabling the automated identification of high-anthocyanin kernels at the single-seed level.

In future research endeavors, employing variable selection techniques could enhance the efficacy of utilizing a comparable data acquisition strategy. Future investigations could explore the impact of various preprocessing methods on the efficacy of models, potentially leading to the development of more robust models. Additionally, there is potential to develop classification models by categorizing anthocyanin content into discrete classes (low, medium, high). This stratification could aid in refining the predictive power of models, facilitating more precise analyses of anthocyanin distribution at the intact kernel level. By systematically investigating these aspects, researchers could significantly advance the field, contributing to both the theoretical understanding and practical application of model development.

## Figures and Tables

**Table 1:** The number of models developed in this study and the size of the input data used.

		The Number of Models		Training Data Size (Row × Column)	
Model	Dataset	Embryo-Up	Embryo-Down	n × p (Embryo-Up)	n × p (Embryo-Down)
Classic (PLSR)	HSV	1	1	181 × 9	185 × 9
	LAB	1	1	181 × 9	185 × 9
	RGB	1	1	181 × 9	185 × 9
	NIR	1	1	181 × 1200	185 × 1200
	RGB+HSV+LAB+NIR	1	1	181 × 1227	185 × 1227
AutoML	HSV	193	192	181 × 9	185 × 9
	LAB	196	191	181 × 9	185 × 9
	RGB	186	191	181 × 9	185 × 9
	NIR	194	203	181 × 1200	185 × 1200
	RGB+HSV+LAB+NIR	180	182	181 × 1227	185 × 1227
	Total	954	964		

**Table 2:** Descriptive statistics for predictive datasets used in embryo-up kernel samples.

Model Dataset	*n*	Mean	STD ^1^	Min	Max
Embryo-Up	181	29.97	34.86	5.53	184.67
Embryo-Down	185	28.82	34.19	5.53	184.63

^1^ STD: Standard deviation.

**Table 3:** Classic (PLSR) and the best model results selected by AutoML for anthocyanin content using datasets.

Side	Model	Dataset	MAE	RMSE	MSE	R^2^	MAPE
Embryo-Up	Classic (PLSR)	HSV	19.00	27.15	737.24	0.39	1.15
		LAB	20.09	28.79	828.79	0.31	1.20
		RGB	20.43	28.82	830.40	0.31	1.47
		NIR	23.81	32.95	1086.00	0.10	1.65
		RGB+HSV+LAB+NIR	19.76	28.58	816.72	0.32	1.17
		Mean	20.62	29.26	859.83	0.29	1.33
	The Best (Ensemble)	HSV	11.38	18.60	346.13	0.71	0.46
		LAB	11.52	20.25	410.24	0.66	0.50
		RGB	10.34	16.37	268.11	0.78	0.52
		NIR	18.35	25.37	643.80	0.47	1.26
		RGB+HSV+LAB+NIR	10.79	18.23	332.46	0.72	0.51
		Mean	12.48	19.77	400.15	0.67	0.65
Embryo-Down	Classic (PLSR)	HSV	16.01	26.24	688.76	0.41	0.77
		LAB	16.45	25.84	667.61	0.43	0.85
		RGB	17.77	27.27	743.50	0.36	1.01
		NIR	21.29	29.16	850.16	0.27	1.57
		RGB+HSV+LAB+NIR	17.03	24.27	589.00	0.49	1.08
		Mean	17.71	26.56	707.81	0.39	1.05
	The Best (Ensemble)	HSV	9.24	16.86	284.18	0.76	0.35
		LAB	9.67	17.92	321.13	0.72	0.39
		RGB	11.45	18.75	351.74	0.70	0.54
		NIR	15.23	21.88	478.57	0.59	0.98
		RGB+HSV+LAB+NIR	10.25	16.60	275.51	0.76	0.50
		Mean	11.17	18.40	342.22	0.71	0.55

## Data Availability

The original contributions presented in the study are included in the article; further inquiries can be directed to the corresponding author.

## Abbreviations

The following abbreviations are used in this manuscript:

RGB	Red–Green–Blue
HSV	Hue–Saturation–Value
NIR	Near-Infrared
PLSR	Partial Least Square Regression
RMSE	Root Mean Squared Error
MAE	Mean Absolute Error
MAPE	Mean Absolute Percentage Error

## Appendix A

**Table A1:** Kernel samples used in the study.

No	Kernel		Kernel		Kernel		Kernel
1		11		21		31	
2		12		22		32	
3		13		23		33	
4		14		24		34	
5		15		25		35	
6		16		26		36	
7		17		27		37	
8		18		28		38	
9		19		29		39	
10		20		30		40	
